# Macrophage Populations in Visceral Adipose Tissue from Pregnant Women: Potential Role of Obesity in Maternal Inflammation

**DOI:** 10.3390/ijms19041074

**Published:** 2018-04-04

**Authors:** Eyerahi Bravo-Flores, Ismael Mancilla-Herrera, Salvador Espino y Sosa, Marco Ortiz-Ramirez, Verónica Flores-Rueda, Francisco Ibargüengoitia-Ochoa, Carlos A. Ibañez, Elena Zambrano, Mario Solis-Paredes, Otilia Perichart-Perera, Maribel Sanchez-Martinez, Diana Medina-Bastidas, Enrique Reyes-Muñoz, Guadalupe Estrada-Gutierrez

**Affiliations:** 1 Department of Immunobiochemistry, Instituto Nacional de Perinatologia, 11000 Mexico City, Mexico; 2 Posgrado en Ciencias Biologicas, Universidad Nacional Autonoma de Mexico, 0451110 Mexico City, Mexico; 3 Department of Infectology and Immunology, Instituto Nacional de Perinatologia, 11000 Mexico City, Mexico; 4 Clinical Research Branch, Instituto Nacional de Perinatologia, 11000 Mexico City, Mexico; 5 Department of Obstetrics, Instituto Nacional de Perinatologia, 11000 Mexico City, Mexico; 6 Department of Reproductive Biology, Instituto Nacional de Ciencias Médicas y Nutricion Salvador Zubiran, 14080 Mexico City, Mexico; 7 Department of Human Genetics and Genomics, Instituto Nacional de Perinatologia, 11000 Mexico City, Mexico; 8 Department of Nutrition and Bioprogramming, Instituto Nacional de Perinatologia, 11000 Mexico City, Mexico; 9 Endocrinology Division, Instituto Nacional de Perinatologia, 11000 Mexico City, Mexico; 10 Research Division, Instituto Nacional de Perinatologia, 11000 Mexico City, Mexico

**Keywords:** pregnancy, obesity, resident macrophage, recruited macrophage, inflammation, visceral adipose tissue

## Abstract

Obesity is associated with inflammatory changes and accumulation and phenotype polarization of adipose tissue macrophages (ATMs). Obese pregnant women have alterations in adipose tissue composition, but a detailed description of macrophage population is not available. In this study, we characterized macrophage populations in visceral adipose tissue (VAT) from pregnant women with normal, overweight, and obese pregestational weight. Immunophenotyping of macrophages from VAT biopsies was performed by flow cytometry using CD45 and CD14 as markers of hematopoietic and monocyte linage, respectively, while HLA-DR, CD11c, CD163, and CD206 were used as pro- and anti-inflammatory markers. Adipocyte number and size were evaluated by light microscopy. The results show that pregnant women that were overweight and obese during the pregestational period had adipocyte hypertrophy. Two different macrophage populations in VAT were identified: recruited macrophages (CD45^+^CD14^+^), and a novel population lacking CD45, which was considered to be a resident macrophages subset (CD45^−^CD14^+^). The number of resident HLA^−^DR^low/−^ macrophages showed a negative correlation with body mass index (BMI). Both resident and recruited macrophages from obese women expressed higher CD206 levels. CD11c expression was higher in resident HLA-DR^+^ macrophages from obese women. A strong correlation between CD206 and CD11c markers and BMI was observed. Our findings show that being overweight and obese in the pregestational period is associated with adipocyte hypertrophy and specific ATMs populations in VAT.

## 1. Introduction

Visceral adipose tissue (VAT) is considered to be an energy storage depot and has now been recognized as a highly active organ responsible for controlling numerous metabolic, hormonal, and immune processes [1,2,3]. Moreover, it is well-accepted that a large VAT mass is a main risk factor for developing metabolic dysfunction [4]. This tissue is constituted mainly by two components: (1) mature adipocytes that are responsible for controlling metabolism by storing excess calories as lipids as well as the production of endocrine hormones, such as leptin, resistin, and adiponectin [5]; and (2) stromal vascular fraction (SVF), which is a rich source of preadipocytes, endothelial cells, fibroblasts, mesenchymal cells, hematopoietic progenitor cells, and immune cells [6]. Of the heterogeneous leukocytes in VAT, macrophages are considered to be important contributors to adipose tissue maintenance, regulating immune and metabolic functions through cross-talk with adipocytes [7].

It has been demonstrated in animal models and human adipose tissue that macrophage phenotype is shaped by the microenvironment in the adipose tissue depots. Adipose tissue macrophage (ATM) accumulation and polarization from the anti-inflammatory (M2) to the pro-inflammatory (M1) phenotype have been associated with the metabolic and inflammatory changes that are the hallmarks of obesity [2,8].

On the other hand, the growing rates of overweight women at reproductive age and the increasing number of overweight or obese women beginning pregnancy are considered to be serious public health issues in the 21st century [9]. This has consequences in the mother and has subsequent effects on the child, leading to obesity, dyslipidemias, Type 2 diabetes, and hypertension later in life [10,11].

The potential role of ATMs in pregnancy as regulators of maternal inflammation is not yet fully understood. Therefore, the aim of this study was to characterize macrophage populations in VAT from pregnant women and to evaluate differences by weight status.

## 2. Materials and Methods

### 2.1. Patients and Donor Selection

Protocol 212250-3210-21002-06-15 was approved by the IRB of the Instituto Nacional de Perinatologia in Mexico City. Each participant signed a written informed consent form.

A cross-sectional study was conducted from January 2016 to December 2016 at Instituto Nacional de Perinatologia, Mexico City, Mexico. Women were recruited consecutively and written informed consent was obtained from each one. During this period, 1294 newborns were delivered by cesarean section at term (37–40 weeks of gestation by obstetric ultrasonography) in our institution. A total of 69 women met the inclusion criteria, 34 declined to participate in the study, it was not possible to obtain samples from 8 women, and 6 collected samples were insufficient for the experiments. Twenty-one healthy pregnant women with intact membranes, no clinical evidence of intrauterine infection, and normal weight gain during pregnancy were enrolled according to the inclusion criteria: age 20–40 years, pregestational Body Mass Index (BMI) >18.5 kg/m^2^ and known pregestational weight. Exclusion criteria included diagnosis of gestational or pre-gestational diabetes, thyroid, hypertensive, or immunological disease, treatment with drugs that could affect metabolism or inflammation during pregnancy, intrauterine growth restriction, or fetal genetic abnormalities. Maternal weight and height at the end of pregnancy were measured by trained personnel. Pregestational weight was collected from clinical files and BMI was calculated according to the World Health Organization criteria. The subjects were stratified into three groups: normal weight (BMI 18.5–24.9 kg/m^2^; *n* = 7), overweight (BMI 25.0–29.9 kg/m^2^; *n* = 7), and obesity (BMI >29.9 kg/m^2^; *n* = 7).

### 2.2. Collection of Visceral Adipose Tissue

Visceral Adipose Tissue biopsies (5 × 5 cm) were obtained from the omentum (middle bottom portion) prior to the hysterotomy for delivery under sterile conditions. Samples were transported in sterile recipients and processed up to 1 h after collection.

### 2.3. Isolation of the Stromal Vascular Fraction from Visceral Adipose Tissue

Visceral Adipose Tissue biopsies were washed in 1× Phosphate Buffered Saline (1× PBS) before being minced with sterile scissors to be digested with collagenase solution (0.01 M PBS, 5 mM glucose, 1.5% *w*/*v* BSA, and 0.25% collagenase type I (GIBCO by Life Technologies, Carlsbad, CA, USA) at a pH of 7.4) for 1 h at 37 °C under constant shaking. After this, the digested tissue was filtered and centrifuged at room temperature (RT) for 10 min at 200× *g* to separate adipocytes by flotation from the stromal vascular fraction. After discarding the supernatant, the cell pellet containing preadipocytes, endothelial cells, and immune cells was incubated in erythrocyte lysis buffer (Roche, Basel, Switzerland) for 5 min at RT. The reaction was stopped by addition of ice-cold 1× PBS before the solution was centrifuged at 4 °C for another 5 min at 1500× *g*. The cell pellet was washed with 1× PBS twice under the same conditions, and cells were counted in a standard hemocytometer using trypan blue staining to assess viability.

### 2.4. Immunostaining for Macrophages from Stromal Vascular Fraction

The stromal vascular fraction cell suspensions (1 × 10^6^ cells/mL) were incubated with the labelled monoclonal antibodies CD45/PE-Cy7 (Cat. 304016; clone HI30), CD14/APC-Cy7 (Cat. 325620; clone HCD14), HLA DR/PE-Cy5 (Cat. 307608; clone L243), CD11c/PE (Cat. 301606; clone 3.9), CD163/FITC (Cat. 333618; clone GHI/61), CD206/APC (Cat. 321110; clone 15-2), and DAPI solution (Cat. 422801) for 15 min at RT in the dark. All antibodies were purchased from BioLegend (San Diego, CA, USA). Appropriate Fluorescent Menus One controls were also used. Cells were fixed using a fluorescence-activated cell sorting (FACS) lysing solution (Cat. 349202, BD Bioscience, Frankling Lakes, NJ, USA) according to the manufacturer’s instructions. Flow cytometry analysis was performed using a FACS Aria III with the FASCDiva software 6.0 (BD Bioscience).

The gating strategy used to identify macrophage subpopulations in the stromal vascular fraction consisted of excluding aggregated and dead cells. After this, cells were gated based on CD45 versus CD14 expression as either CD45^−^CD14^+^ (resident macrophages) or CD45^+^CD14^+^ (recruited macrophages). The HLA-DR, CD11c, CD163, and CD206 expression was determined as the median fluorescence intensity (MFI) for each marker.

### 2.5. Hematoxylin and eosin stain of Adipose Tissue Sections

Paraffin-embedded 5-µm VAT sections fixed in 10% formaldehyde were deparaffinized, rehydrated, stained with hematoxylin and eosin, and mounted using a DPX mounting medium (Sigma-Aldrich, St. Louis, MO, USA). A minimum of four randomly chosen low-power (20× fields were counted by the same observer to determine adipocyte number and size per field in VAT using the AxioVision version 4.9.1 Microscopy Software (Zeiss, Oberkochen, Germany).

### 2.6. Statistical Analysis

Distribution analysis was assessed using the Shapiro–Wilk normality test. Multiple groups were analyzed by one-way ANOVA or Kruskal–Wallis one-way analysis of variance, while pairs of groups were compared by the Mann–Whitney U test as appropriate for variables that were not normally distributed. The relationship between two variables was determined by Spearman’s rank correlation coefficient. A linear regression model was performed to evaluate the effect of BMI on the variables of interest. Statistical analyses were performed with the IBM (Armonk, NY, USA) SPSS 20.0 statistical software. Unless otherwise stated, all data were expressed as mean ± SD (standard deviation) or median and interquartile range. In the present study, *p <* 0.05 was considered statistically significant.

## 3. Results

### 3.1. Pregestational Obesity Is Associated with Visceral Adipocyte Hypertrophy

The characteristics of the studied population are shown in Table 1. Patients did not differ in maternal age, parity, and fasting glucose. Overweight and obese women delivered newborns with higher weight and height compared to the normal group (*p* = 0.03 and *p* = 0.02, respectively, data not shown), while no differences were found between gender, Capurro, Apgar, and Silverman–Anderson tests.

**Figure 1:**
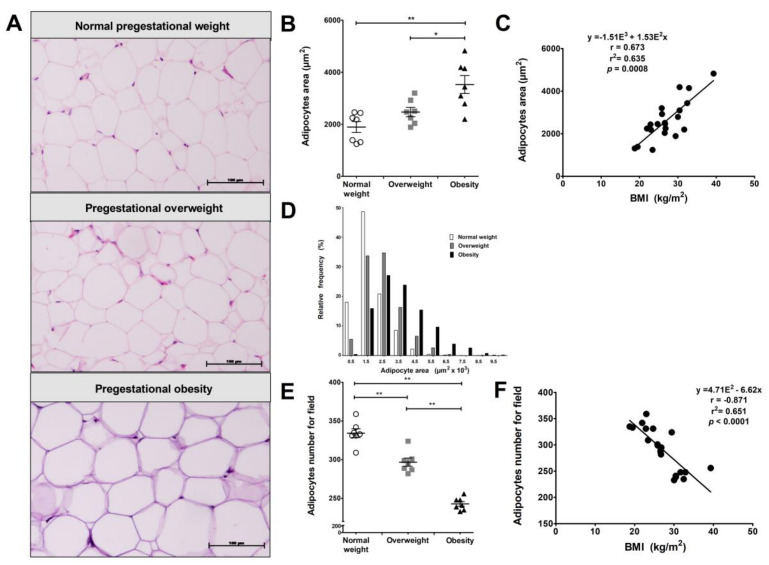
Adipocyte hypertrophy in visceral adipose tissue (VAT) from obese pregnant women. (**A**) H&E staining of VAT from pregnant women with normal pregestational weight (BMI < 25.0), overweight (25.0 ≤ BMI < 30.0) and obesity (BMI ≥ 30.0) with scale bars of 100 µm and 20× magnification. The representative images show larger size adipocytes in the overweight and obese groups. (**B**) Scatter plot of adipocyte size (area per adipocyte) in VAT from normal pregestational weight (*n* = 7), overweight (*n* = 7), and obese (*n* = 7) subjects showing significantly larger adipocytes in the obese group. (**C**) Relative frequencies of adipocytes per area in the study groups. (**D**) Scatter plot of adipocyte number per four 20× fields show a significant decrease in the overweight and obese groups. (**E**,**F**). Data are expressed as median of adipocyte size and number per field. There is a significant correlation between adipocyte size and number with BMI in VAT (*n* = 21). * *p* < 0.05 and ** *p* < 0.01, calculated by Mann–Whitney test.

Visceral adipocyte size was measured in these patients. Adipocyte hypertrophy is observed both in VAT from women with a pregestational obesity or overweight status (Figure 1A). Adipocyte size in obese women was significantly bigger compared to women with normal weight (1.5–8.5 µ^2^ × 10^3^ versus 0.5–5.5 µ^2^ × 10^3^, *p* = 0.004). On the other hand, the overweight group tended to reach similar size to those from the normal group (0.5–6.5 µ^2^ × 10^3^ versus 0.5–5.5 µ^2^ × 10^3^, *p* > 0.05 (Figure 1B), with a positive correlation observed between the adipocyte size and BMI (Figure 1C). Additionally, obese women showed an increased dispersion of adipocyte size compared to normal and overweight women, while the data dispersion of overweight was greater than normal weight women (Figure 1D). Adipocyte number per four fields was compared between the study groups, showing that women with pregestational obesity or overweight status have fewer adipocytes per field compared with the normal weight group (*p* = 0.01; Figure 1E). We found an association between adipocyte size and number with BMI, while the linear regression model predicts that an increased BMI explains up to 63.5% of the variance in adipocyte size and up to 65.1% of the decrease in adipocyte number per field (Figure 1F).

### 3.2. Resident and Recruited Macrophage Subsets Are Present in Visceral Adipose Tissue from Pregnant Women

Multiparametric analysis with CD45 and CD14 revealed two different cell populations in the VAT stromal vascular fraction: one with hematopoietic origin (CD45^+^CD14^+^) and one with non-hematopoietic origin, which was characterized by the absence of CD45 marker (CD45^−^CD14^+^).

**Figure 2:**
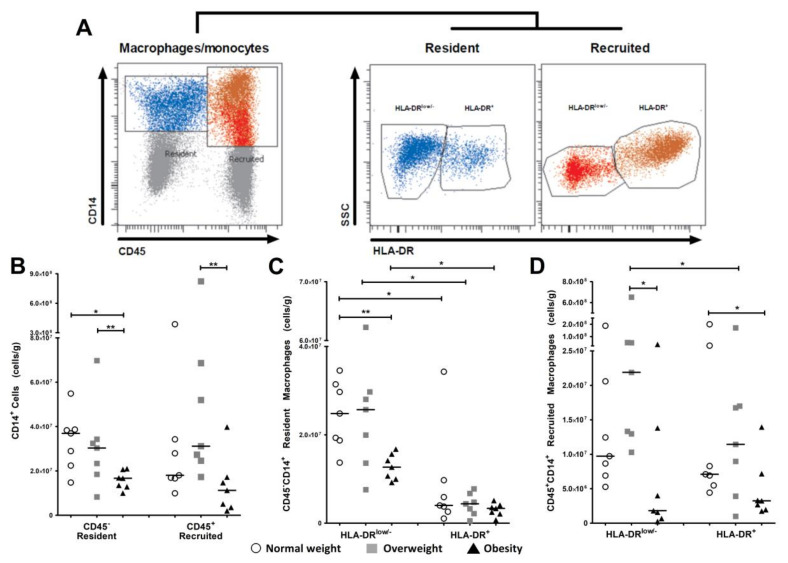
Resident and recruited macrophages subsets are present in VAT from pregnant women. Immunophenotyping of stromal vascular fraction macrophages obtained from VAT collected during cesarean section of women who started pregnancy with normal weight or who were overweight or obese using flow cytometry. (**A**) After exclusion of doublet cells and death cells, a CD45 versus CD14 gate identified two different cell populations: one CD45^+^CD14^+^ population with hematopoietic origin (recruited) and another CD45^−^CD14^+^ population with non-hematopoietic origin (resident). Both populations were gated based on macrophage marker HLD-DR on total CD14-positive cells. Two subsets were identified in resident and recruited macrophages: HLA-DR^low/−^ and HLA-DR^+^. Plots illustrate representative data from individual subjects. (**B**) Scatter plots showing cell number per gram of resident and recruited CD14^+^ cells in pregnant women with normal pregestational weight (*n* = 7), overweight (*n* = 7), and obesity (*n* = 7). Data show that resident cell number (CD45^−^CD14^+^) is lower in obese subjects, while the number of recruited cells (CD45^+^CD14^+^) is higher in women who are overweight in the pregestational period. (**C**,**D**) VAT macrophage subsets in resident and recruited cells. Scatter plots show a higher number of resident macrophages with HLA-DR^low/−^ expression compared to HLA-DR^+^ macrophages in all study groups, while the number of CD45^−^CD14^+^ HLA-DR^low/−^ is significantly lower in the pregestational obesity group. In the recruited macrophage population, both subsets (CD45^−^ CD14^+^ HLA-D^low/−^ and CD45^−^CD14^+^ HLA-DR^+^) are significantly decreased in the pregestational obesity group. Recruited HLA-DR^low/−^ macrophages are significantly higher in the overweight group compared with recruited HLA-DR^+^ macrophages. Data are expressed as median of cells/g. * *p* < 0.05 and ** *p* < 0.01, calculated by Mann–Whitney test.

In addition, based on the HLA-DR expression, four subpopulations were clearly identified: (i) CD45^−^CD14^+^HLA-DR^low/−^; (ii) CD45^−^CD14^+^HLA-DR^+^; (iii) CD45^+^CD14^+^HLA-DR^low/−^; and (iv) CD45^+^CD14^+^HLA-DR^+^. The two CD45^−^CD14^+^ cell populations were classified as resident macrophages subsets, while double-positive cell populations (CD45^+^CD14^+^) were described as recruited macrophages subsets (Figure 2A).

The resident cell number present in VAT was significantly lower in women with pregestational obesity compared to both overweight (*p* < 0.01) and lean women (*p* < 0.05), while the recruited cell number showed a significant reduction (*p* < 0.01) in the obese group compared to the overweight group. However, this did not differ for women who started their pregnancy with normal weight (*p* > 0.05; Figure 2B).

The analysis between VAT macrophage subsets demonstrated that there was a higher number of resident HLA-DR^low/−^ macrophages in all study groups compared to the HLA-DR^+^ resident macrophage subset (*p* < 0.05). Furthermore, data provided evidence that women with pregestational obesity had a significant reduction (*p* < 0.01) in the number of resident HLA-DR^low/−^ macrophages compared with the normal weight group (Figure 2C).

On the other hand, the analysis in the recruited subsets showed that the number of HLA-DR^low/−^ macrophages was higher compared to the number of HLA-DR^+^ macrophages in the overweight group (*p* < 0.05). No differences in the proportion of recruited macrophage subsets were found in the other study groups. The intergroup analysis demonstrated that obese women had significantly lower recruited HLA-DR^low/−^ and HLA-DR^+^ macrophages compared to overweight and lean women, respectively (*p* < 0.01; Figure 2D).

### 3.3. Resident HLA-DR^low/−^ Macrophage Subset Is Influenced by Body Mass Index

**Figure 3:**
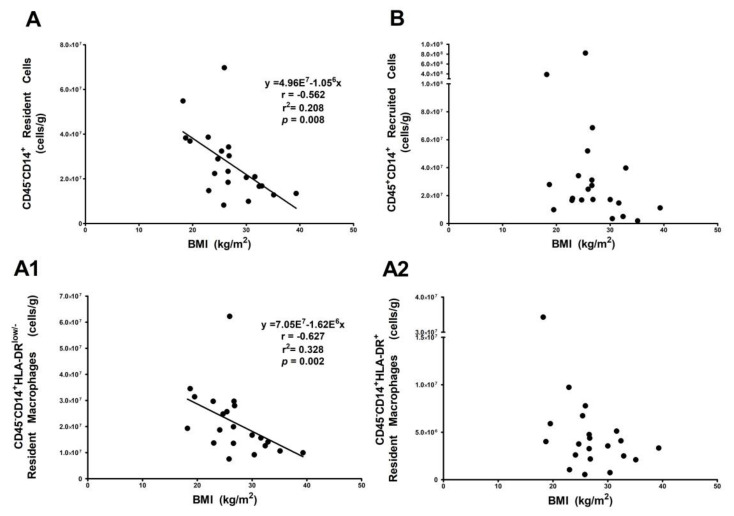
Resident HLA-DR^low/−^ macrophage subset decreases with BMI. Spearman correlation between (**A**) resident and (**B**) recruited CD14^+^ cells as well as (**A1**,**A2**) resident macrophage subsets. The number of resident cells (CD45^−^CD14^+^) (**A**) and CD45^−^ CD14^+^HLA-DR^low/−^ (**A1**) cells decreases as BMI increases (*p <* 0.01, *r* = 0.627; *p* < 0.01, *r* = 0.562).

Associations between VAT macrophage populations and BMI were determined. A linear regression model showed that the total number of resident cells per gram of VAT decreased by 32.8% with an increase in BMI (Figure 3A). However, this finding was not observed in the recruited macrophages (Figure 3B). Moreover, a linear regression analysis was performed to evaluate the effect of BMI on cell number for each resident macrophage subset, which revealed that higher BMI values explained up to 20.8% of variance in the number of resident HLA-DR ^low/−^ macrophages, although this did not explain the HLA-DR^+^ macrophage subset (Figure 3A1,A2).

### 3.4. CD206 and CD11c Surface Expression Is Higher in Recruited and Resident Macrophages from Women with Pregestational Obesity

**Figure 4:**
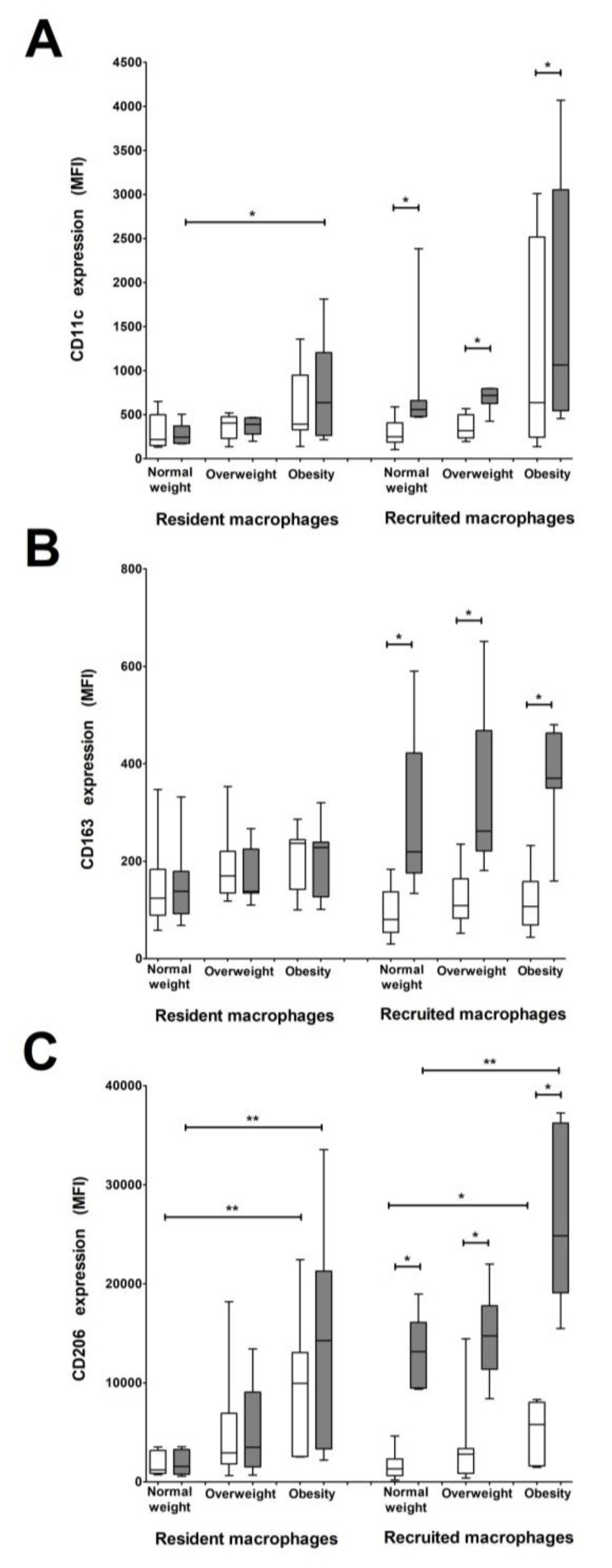
Resident and recruited macrophages subsets show differential CD11c, CD163, and CD206 expression depending on pregestational BMI. (**A**–**C**) Bars show surface expression of CD11c (**A**), CD163 (**B**), and CD206 (**C**) in resident and recruited macrophage subsets: HLA-DR^low/−^ (white) and HLA-DR^+^ (grey) in VAT from normal weight (*n* = 7), overweight (*n* = 7), and obese (*n* = 7) pregnant women. CD11c expression is higher in resident and recruited HLA-DR^+^ macrophages from women with pregestational obesity, which increases with BMI only in the recruited population. CD163 expression is higher in recruited HLA-DR^+^ macrophages and the expression in this subset increases with BMI. CD206 expression is higher in both resident macrophage subsets in the obese group, and the expression in the recruited subsets increases with BMI. Data are expressed as median fluorescence intensity (MFI). * *p* < 0.05 and ** *p* < 0.01, calculated by Mann–Whitney test.

To further characterize macrophage populations in VAT, the expression of CD11c, CD163, and CD206 surface markers was determined. Resident HLA-DR^+^ macrophages exhibited a higher expression of CD11c in obese women compared to women with normal pregestational weight, while recruited HLA-DR^+^ macrophages showed a higher CD11c expression in all study groups compared to recruited HLA-DR^low/−^ macrophages (Figure 4A).

A higher level of CD163 expression was found in recruited HLA-DR^+^ macrophages in all study groups compared to the HLA-DR^low/−^ macrophage subset. No differences in CD163 expression were found between the resident macrophage subsets in any of the study groups (Figure 4B).

Recruited HLA-DR^+^ macrophages from every study group expressed a higher intensity of CD206 than HLA-DR^low/−^ macrophages, showing a trend to a higher expression with an increase in BMI. There was a significant difference between normal weight and obesity (*p* < 0.01). These differences in CD206 intensity were also found between the obese and normal weight groups for resident HLA-DR^+^ macrophages (*p* < 0.01). Moreover, HLA-DR^low/−^ resident and recruited macrophage subsets had higher CD206 expression in the obese group than in the normal pregestational weight group (*p* < 0.01 and *p* < 0.05, respectively; Figure 4C).

A correlation analysis between the surface markers in the macrophage cell subsets and BMI was performed, showing that CD11c expression in resident HLA^−^DR^+^ macrophages significantly increases with BMI (*r* = 0.6, *p* < 0.01). CD206 expression also increases with BMI in all subsets, with higher ratios in HLA-DR^+^ resident and recruited macrophages (*r* = 0.7, *p* < 0.001) than in HLA-DR^low/−^ subsets of resident and recruited macrophages (*r* = 0.6, *p* < 0.01 and *r* = 0.5, *p* < 0.05, respectively; Table 2).

## 4. Discussion and Conclusions

In this study, we have shown the presence of CD45^−^CD14^+^ macrophage cells in VAT obtained from pregnant women for the first time. Furthermore, we demonstrated that being overweight and obese in the pregestational period is associated with adipocyte hypertrophy and specific ATM populations. The resident HLA-DR^low/−^ macrophage number is significantly decreased in women with pregestational obesity compared to normal weight. On the other hand, we described a positive correlation between CD206 expression and BMI in all macrophage subsets as well as an increased CD11c expression associated with obesity in recruited HLA-DR^+^ macrophages.

Analysis of markers CD45 and CD14 used to identify monocyte/macrophage linage cells in VAT revealed the typical CD45^+^CD14^+^ population with a hematopoietic origin and a newly described CD45^−^CD14^+^ cell population that we propose to be cells with a non-hematopoietic origin. These findings are similar to other reports of non-hematopoietic origin macrophages in different tissues. [12,13,14,15,16]. Moreover, we were able to define two subsets of recruited macrophages (CD45^+^CD14^+^HLA-DR^low/−^ and CD45^+^CD14^+^HLA-DR^+^) that originate from circulating monocytes and are best known as monocyte-derived macrophages. We also defined two subsets of CD45^−^ cells (CD45^−^CD14^+^HLA-DR^low/−^ and CD45^−^CD14^+^HLA-DR) that may be those described as resident macrophages in adipose tissue [17,18]. Our results support the statement that these cells are independent of hematopoietic linage and are crucial for homeostasis and tissue immunity in several tissues, including adipose tissue [19].

There are several hypotheses about the origin of resident macrophages. Hoeffel and Ginhoux proposed that resident macrophages may derive from hematopoietic stem-cell-independent embryonic precursors [20]. Other authors have concluded from in vitro animal models that these cells could have come from a population of preadipocytes with a macrophage-like phenotype [21] or that they have developed in adipose tissue from preadipocytes given their macrophage-like features, such as phagocytic activity, microbicide activity, and expression of some macrophage antigens [22].

We observed differences in the distribution of resident and recruited macrophages among the study groups. Tissue-resident macrophages have a role in the maintenance of white adipose tissue, acting as a link between adipocytes and immune cells to coordinate tissue remodeling and function of white adipocytes [23]. As a consequence, the lower number of resident macrophages found in women with pregestational obesity compared to normal weight in our study could be associated with a loss in tissue maintenance and energy homeostasis in VAT.

Several studies have shown that the HLA-DR marker is associated with immunoregulatory functions in different pathologies, suggesting that antigen DR expression reflects the macrophage activation degree. A low DR expression indicates suppressed macrophage activation [24], while a high DR expression reflects activation of the inflammatory NF-KB pathway [25]. Based on this evidence, it seems reasonable that macrophage populations expressing HLA-DR^+^ and HLA-DR^low/−^ are inflammatory and non-inflammatory macrophages, respectively.

Some authors propose that the imbalance towards an inflammatory environment in obese adipose tissue may result from an accumulation of pro-inflammatory macrophages that originated either locally or were recruited from the circulation [26,27,28,29]. In our study, the lower number of resident HLA-DR^low/−^ macrophages found in women with pregestational obesity compared to normal women could reflect a loss of adipose tissue homeostasis, which favors a pro-inflammatory environment. Additionally, the lower number of resident HLA-DR^low/−^ macrophages strongly correlates with a gradual increase in pregestational BMI, which supports our hypothesis.

Although we also found significant differences in the recruited populations among the study groups, our results suggest that pregestational BMI has a bigger influence on the number of resident cells. Therefore, the changes observed in obese adipose tissue may be due to the decreased number of cells with regulatory functions. Despite resident HLA-DR^low/−^ macrophages possibly having anti-inflammatory properties, our study design did not allow us to fully determine these characteristics and ongoing studies in our laboratory are focused on further characterizing macrophage population functionality in VAT.

The macrophage phenotype has been described as a spectrum from classically activated macrophages (M1) to alternatively activated macrophages (M2). Studies in mice models showed that early stages of adipose tissue expansion are characterized by M2-polarized macrophages with progressive lipid accumulation promoting M1 polarization [30]. CD11c is an integrin used as a monocyte/M1 macrophage activation marker. This marker is increased in peripheral monocytes from patients with hyperlipidemia, which plays an important role in the development of atherosclerosis. Some reports have focused on the role of CD11c in adipose tissue, showing that pro-inflammatory adipose tissue macrophages expressing high CD11c levels are associated with inflammation and insulin resistance [31,32]. In our study, women with a high pregestational BMI (≥30) expressed elevated CD11c levels in resident HLA-DR^+^ macrophages, suggesting that the inflammatory profile observed in obese pregnant women is elicited mainly from resident cells.

On the other hand, alternative macrophages that classically express high CD206 levels have been inversely associated with metabolic disturbances and seem to be present mainly in healthy adipose tissue expansion [33]. A subpopulation expressing CD206^+^CD11c^+^ was associated with obesity and insulin resistance [31]. Interestingly, we found that both resident and recruited macrophages in VAT from women with high BMI expressed high CD206 levels in our study, particularly the recruited HLA-DR^+^ macrophage subpopulation from women with pregestational obesity. This has not been previously reported.

Some authors speculate that in order to control adipose tissue inflammation, M1 macrophages switch activation to an anti-inflammatory phenotype. As observed in our study, pro-inflammatory macrophage subsets also express high levels of CD206 and CD163 markers that usually characterize M2 macrophages, which could be consistent with the hypothesis that macrophages could reverse their phenotype and function depending on the microenvironment [34].

It is widely known that obesity promotes robust changes in adipose tissue morphology, including adipocyte hypertrophy and hyperplasia, as well as changes in the composition of immunological cells [4]. This adipocyte hypertrophy has been associated with adipokine production, cell death, and local adipose hypoxia that contributes to macrophage accumulation and promotes inflammation in adipose tissue [35]. However, most of these studies have been performed in animal models using an obesogenic diet or in human morbid obesity, overlooking the impact of maternal obesity on human reproductive health and early development [36,37,38,39,40,41].

In the present study, we describe the morphologic features of adipocytes present in VAT from pregnant women. We were able to determine a direct correlation with changes in size and number depending on pregestational weight. Our results show that VAT adipocyte hypertrophy and reduction in the number of these cells at the end of pregnancy correlate with being overweight or obese in the pregestational period. It is important to note that in the overweight state, there is moderated hypertrophy as the severity of this condition increases with BMI. Adipocyte hypertrophy may be associated with a higher degree of inflammation in these groups of women. This may lead to adverse perinatal outcomes, such as glucose intolerance and insulin resistance, as has been demonstrated in previous reports using pregnant mice models [42,43]. Our findings are similar to those reported by Haghiac et al., which described that adipocytes from obese pregnant women are larger than those from lean pregnant women, although these authors analyzed abdominal subcutaneous fat [44]. Regarding animal models, Zhang et al. 2011 showed that adipocytes obtained from subcutaneous and parametrial adipose tissue in lean pregnant mice are larger than those from lean non-pregnant mice, concluding that adipocyte hypertrophy is dependent on pregnancy [45]. The strong correlation of BMI with adipocyte size and number found in our study suggests that hypertrophy depends on weight rather than pregnancy state, even though we did not evaluate non-pregnant women.

**Figure 5:**
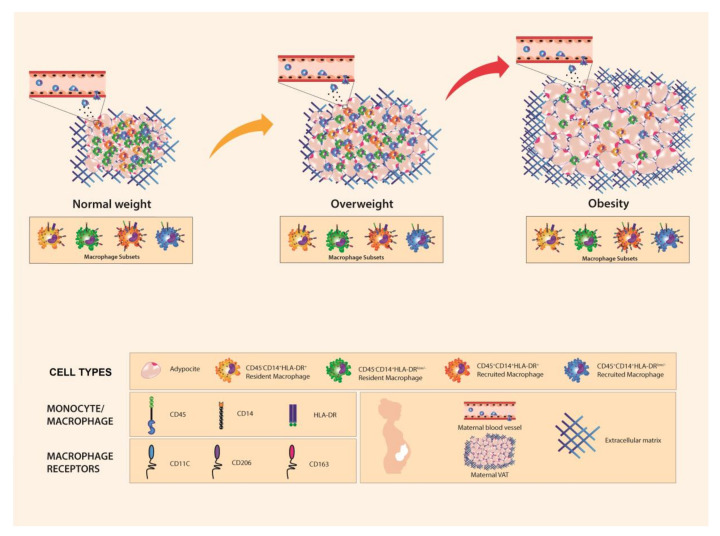
Differences in adipocyte and macrophage subsets in visceral adipose tissue from pregnant women by pregestational weight status. Visceral adipose tissue in pregnant women exhibits differences in adipocyte size and number depending on pregestational BMI. We found that this tissue is composed by two different macrophage populations based on the presence of CD45 and CD14 markers: resident macrophages (CD45^−^CD14^+^) and recruited macrophages (CD45^+^CD14^+^). Furthermore, four subsets were identified based on HLA-DR expression: CD45^−^CD14^+^HLA-DR^low/−^, CD45^−^CD14^+^HLA-DR^+^, CD45^+^CD14^+^HLA-DR^low/−^, and CD45^+^CD14^+^HLA-DR^+^. Macrophages express different levels of CD11c, CD206, and CD163 surface markers. In particular, CD206 increases expression in all macrophage subsets at higher BMI, while CD11c expression only increases in resident HLA-DR^+^ macrophages at higher BMI. Visceral adipose tissue from women with pregestational obesity presents adipocyte hypertrophy and a lower number of resident HLA-DR^low/−^ cells compared to women with normal pregestational weight.

Human VAT is constituted by adipocyte and non-adipocyte cells, such as endothelial cells, fibroblasts, preadipocytes, and several immune cells, such as macrophages [46]. It has been demonstrated that cross-talk is established between them and regulates tissue functionality at different levels [47,48]. Moreover, macrophages seem to play a leading role in the inflammatory response elicited by this tissue in obesity [6,49]. Some studies have characterized macrophage populations and composition using different sources of adipose tissue, which is mostly related to extreme degrees of obesity. This showed that a high infiltration of pro-inflammatory macrophages, especially in VAT, strongly correlates with a local and systemic inflammatory environment [50,51,52,53]. Haghiac et al. reported a higher number of macrophages in subcutaneous adipose tissue from obese pregnant women compared to lean pregnant women, which was correlated with an inflammatory state and higher release of cell-free DNA in the obese group [44]. However, this study lacks a characterization of macrophage populations and does not include an overweight women group as a previous state to obesity. All findings are summarized in Figure 5.

It is important to consider that this macrophage characterization was conducted only at the resolution of pregnancy due to tissue availability and it is not possible to establish if pregnancy itself has any influence on the changes in macrophage subsets. In addition, our experimental design did not allow us to determine the origin and functionality of resident macrophages in human VAT, so further research should focus on this premise.

Overall, these novel findings suggest that adipose tissue inflammation in women that are overweight or obese in the pregestational period is strongly associated with changes in the proportion of the macrophage subpopulations and differences in the expression of anti- and pro-inflammatory surface markers in these cells. According to our results, the first changes could have occurred in the resident macrophages in these two groups of patients, which decrease the number and expression of higher levels of pro- and anti-inflammatory markers. Recruited cells also seem to show differences, particularly the CD45^+^CD14^+^HLA-DR^+^ subset that expresses high levels of anti-inflammatory markers as BMI increases. We infer that these events may occur in order to compensate for the inflammatory process and maintain tissue homeostasis. Leading with this, it would be interesting to explore whether tissue inflammation is more severe in women with excessive weight gain during pregnancy as well as characterize the composition and distribution of macrophage subsets and their potential association with the onset of metabolic diseases during pregnancy and later in the mother and offspring.

## Figures and Tables

**Table 1:** Clinical and demographic characteristics of the population.

	Normal Weight *n* = 7	Overweight *n* = 7	Obesity *n* = 7	*p*
Mother characteristics				
Maternal age (years)	27.6 ± 6.5	32.1 ± 8.3	32.8 ± 4.3	0.286
Pregestational BMI (kg m^−2^)	21.6 ± 2.7	26.7 ± 1.2	33.3 ± 3.4	<0.0001
Parity	3.1 ± 1.4	3.1 ± 1.4	3.5 ± 1.9	0.859
Fasting glucose (mg/dL)	79.4 ± 6.3	78.1 ± 11.0	83.3 ± 8.5	0.498
Newborn characteristics				
Gender	2F/5M	4F/3M	7F/0M	
Birth weight (g)	2868.6 ± 248.9	3408.4 ± 647.3	3363.1 ± 468.1	0.092
Birth height (cm)	47.0 ± 1.6	50.2 ± 2.5	49.1 ± 1.4	0.014
Gestational age (weeks)	37.9 ± 0.8	38.6 ± 1.0	38.5 ± 0.5	0.168
Capurro method (weeks)	38.8 ± 0.7	39.1 ± 1.1	38.2 ± 1.4	0.250
Apgar 1st min (score)	8.0 ± 0.0	8.0 ± 0.5	7.9 ± 0.4	0.741
Apgar 5th min (score)	9.0 ± 0.0	9.0 ± 0.0	9.0 ± 0.0	1.0
Silverman–Andersen	1.6 ± 0.5	1.3 ± 0.5	1.5 ± 1.1	0.357

Data are reported as mean ± standard deviation (SD). BMI = Body Mass Index; F = Female; and M = Male. The *p* values were derived using one-way ANOVA.

**Table 2:** Spearman correlation between markers on macrophage subsets and pregestational BMI.

		BMI			
		Resident		Recruited	
		HLA-DR^low/−^	HLA-DR^+^	HLA-DRl^ow/−^	HLA-DR^+^
**MFI CD11c**	***r***	0.4014	**0.5528**	0.3800	0.4001
	***p***	(*ns*)	(**0.009**)	(*ns*)	(*ns*)
**MFI CD163**	***r***	0.2605	0.2726	0.0474	0.2449
	***p***	(*ns*)	(*ns*)	(*ns*)	(*ns*)
**MFI CD206**	***r***	**0.6255**	**0.6665**	**0.4651**	**0.7197**
	***p***	(**0.002**)	(**0.001**)	(**0.034**)	(**0.0002**)

Data are reported as Spearman’s correlation coefficient. BMI = Body Mass Index; MFI = median fluorescence intensity; *r* = Spearman’s rho; and *ns* = non-significant.
